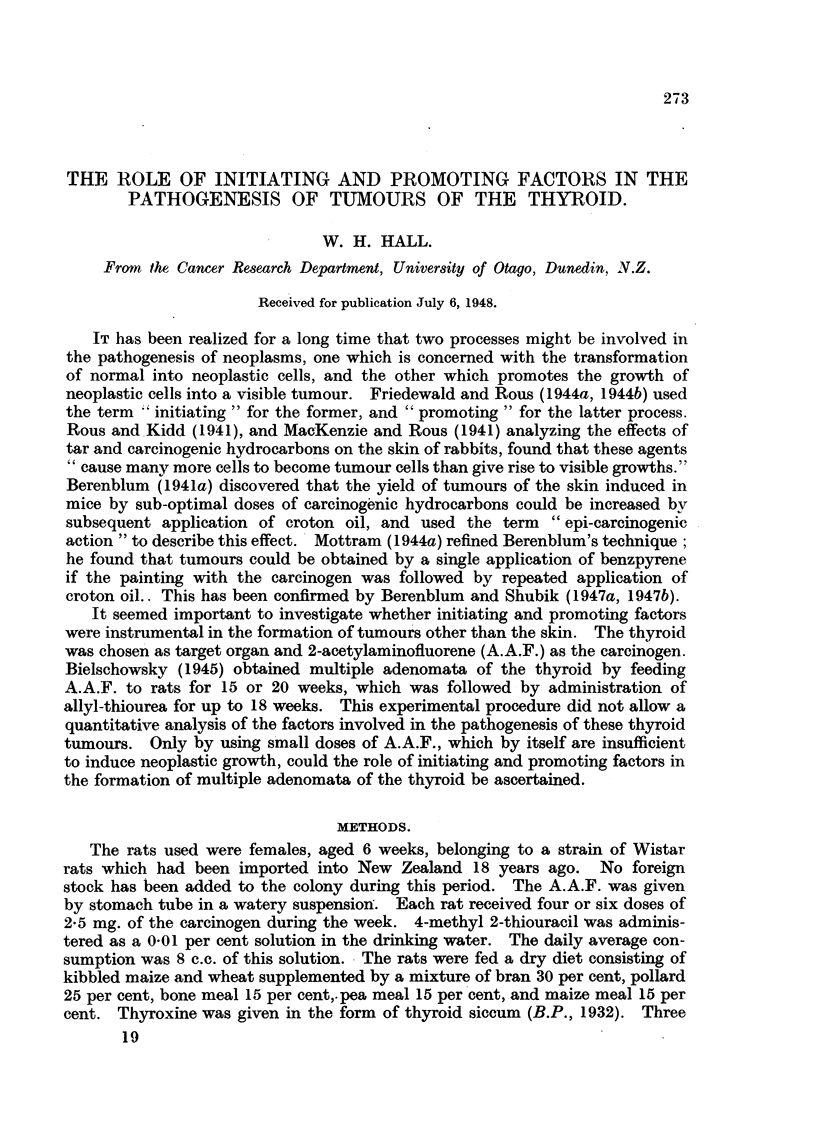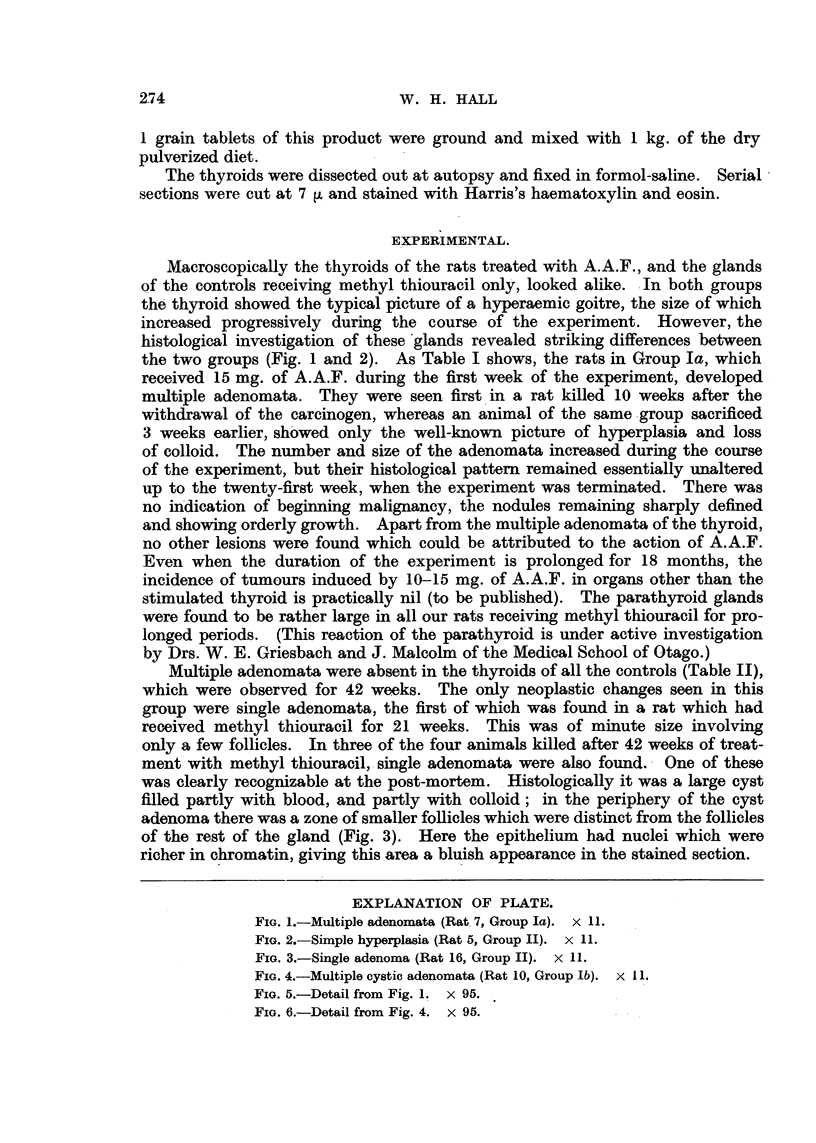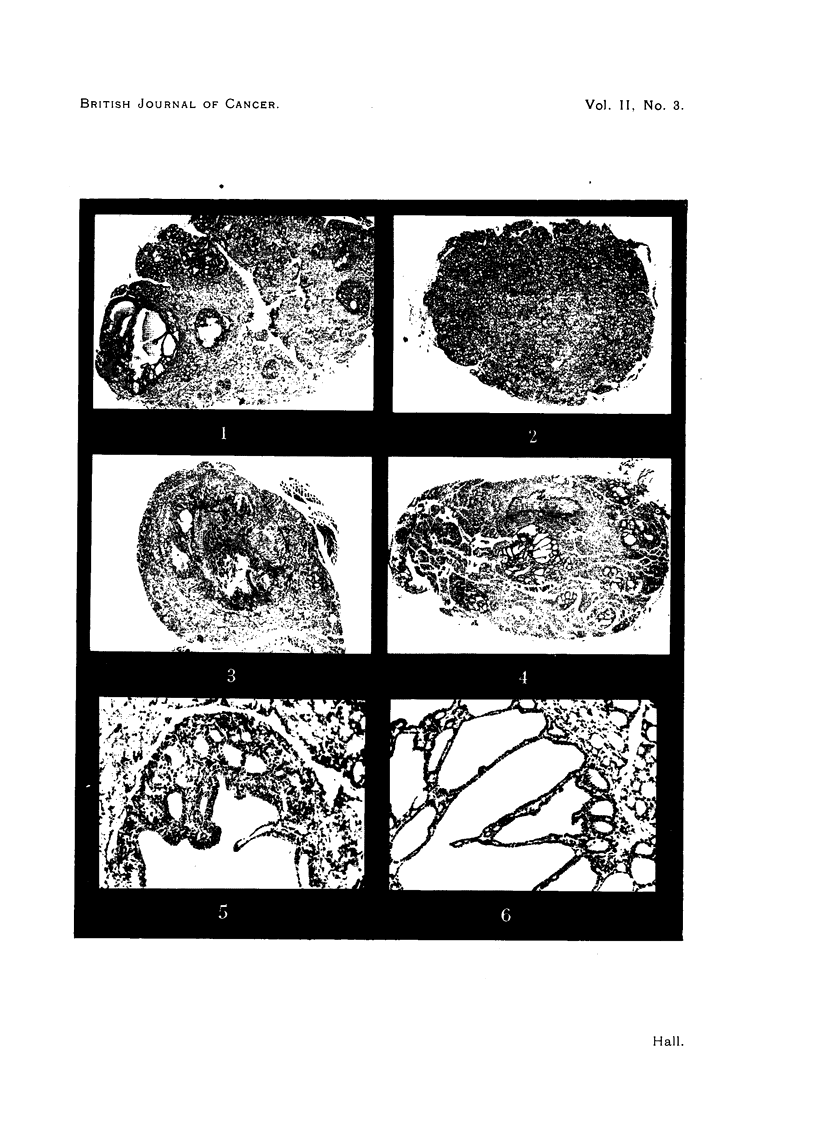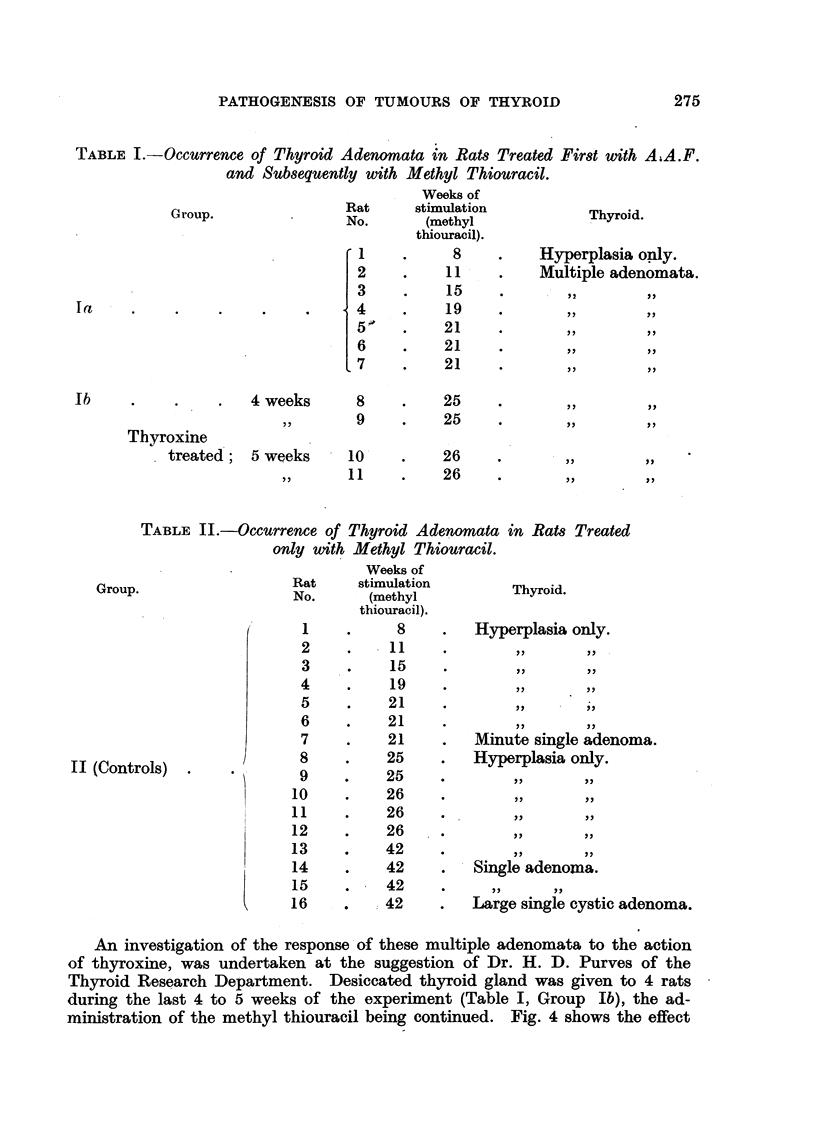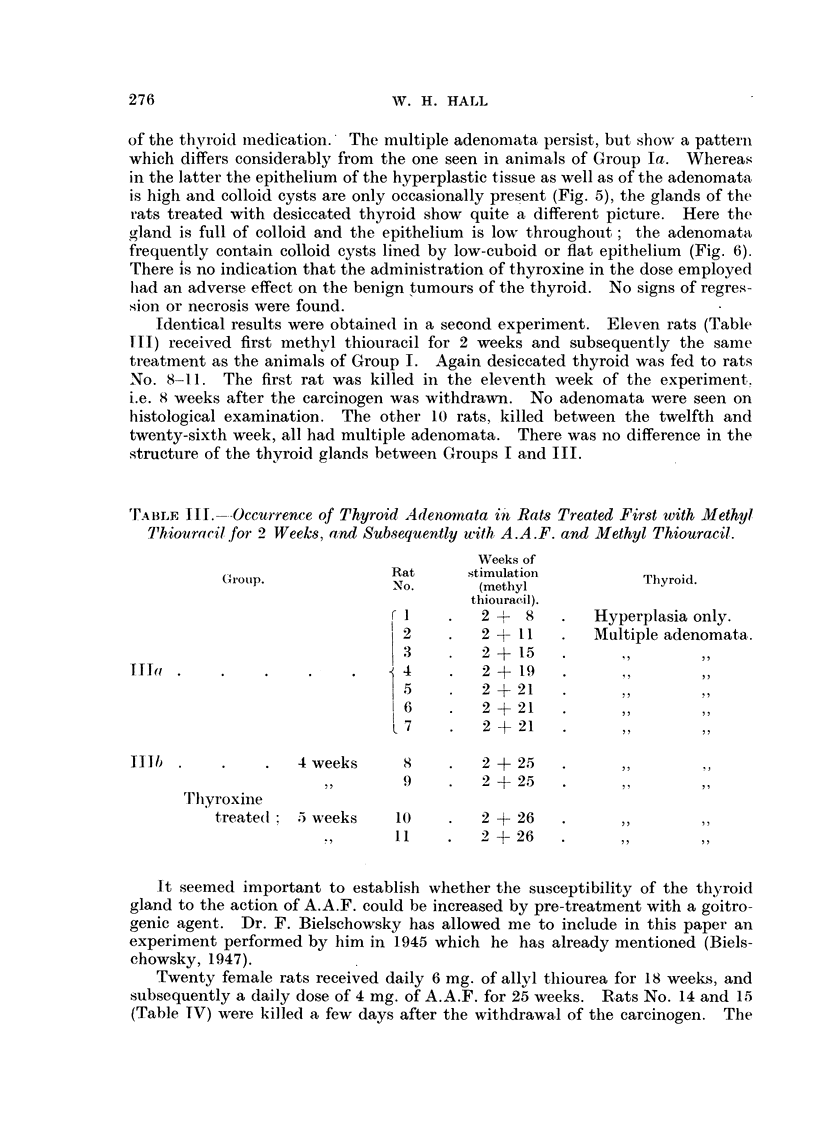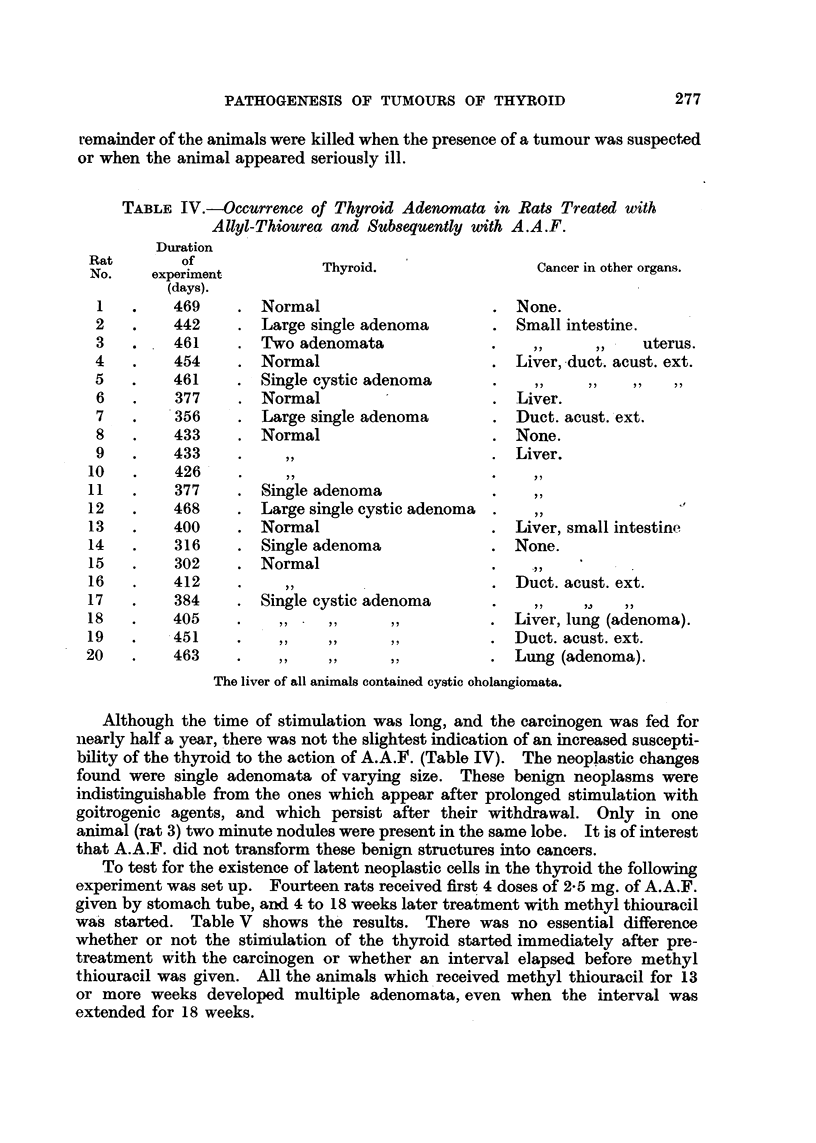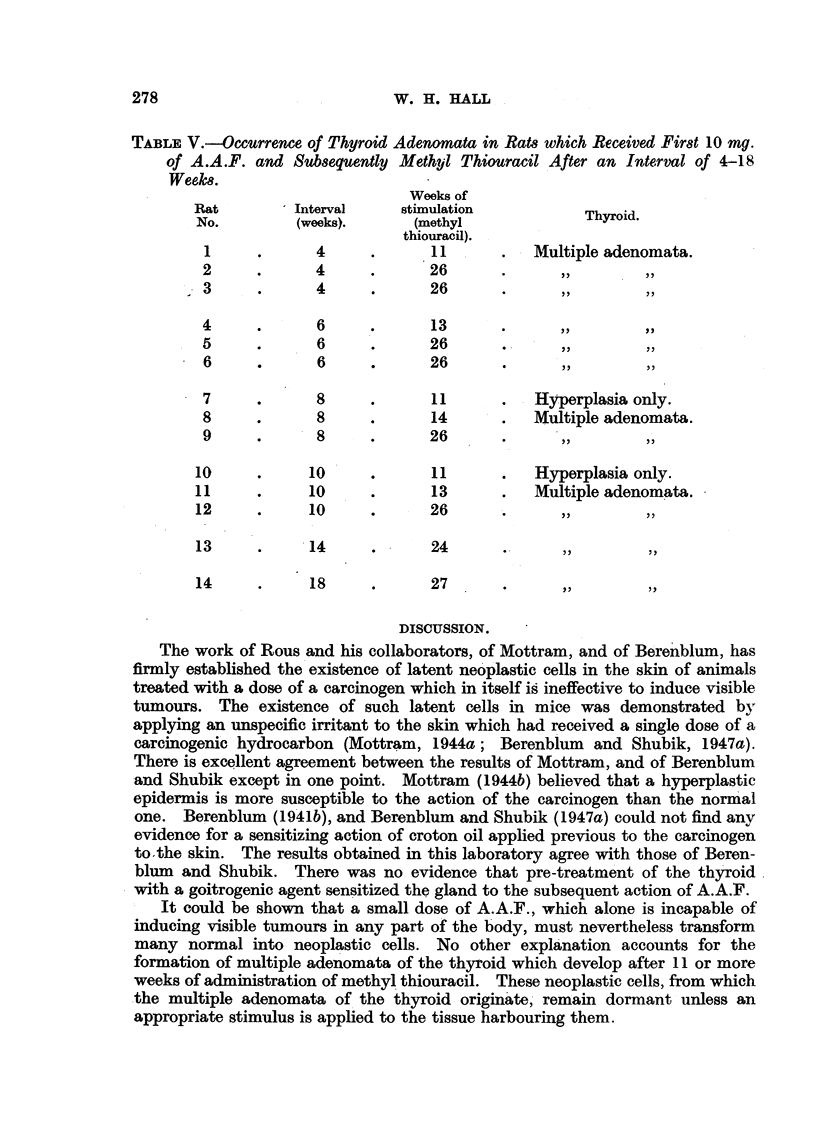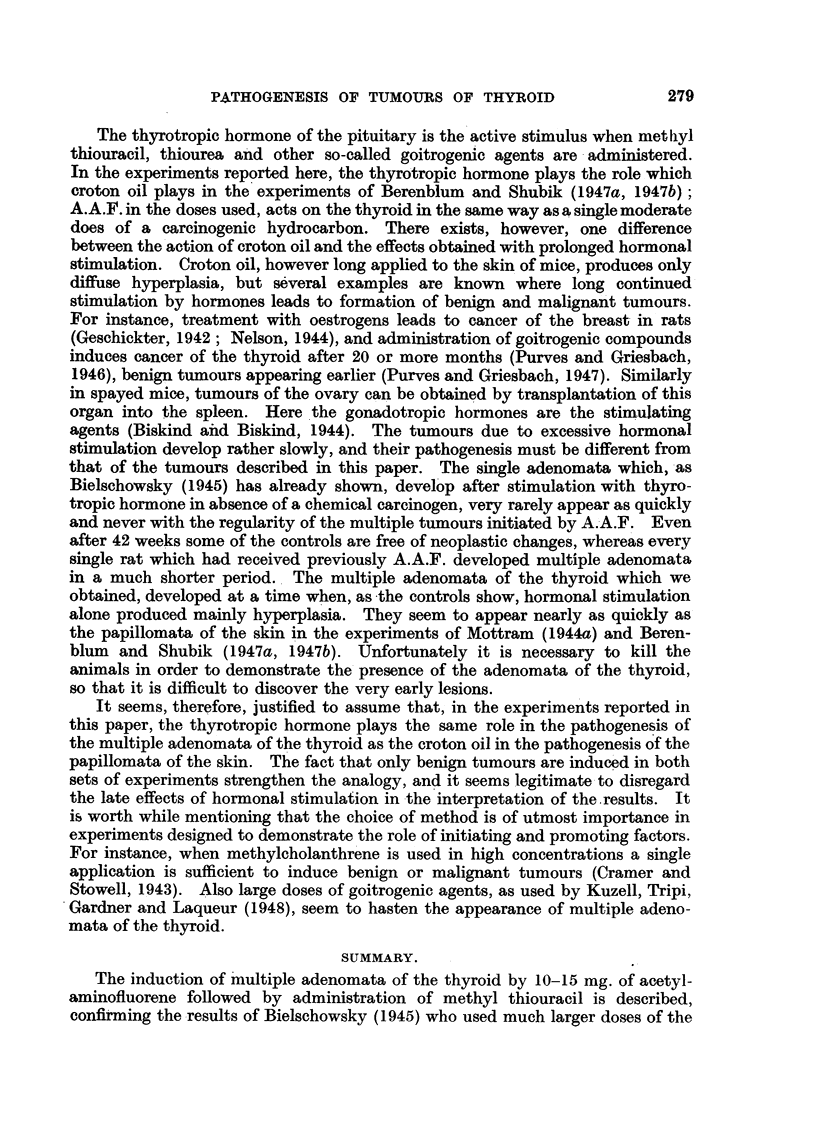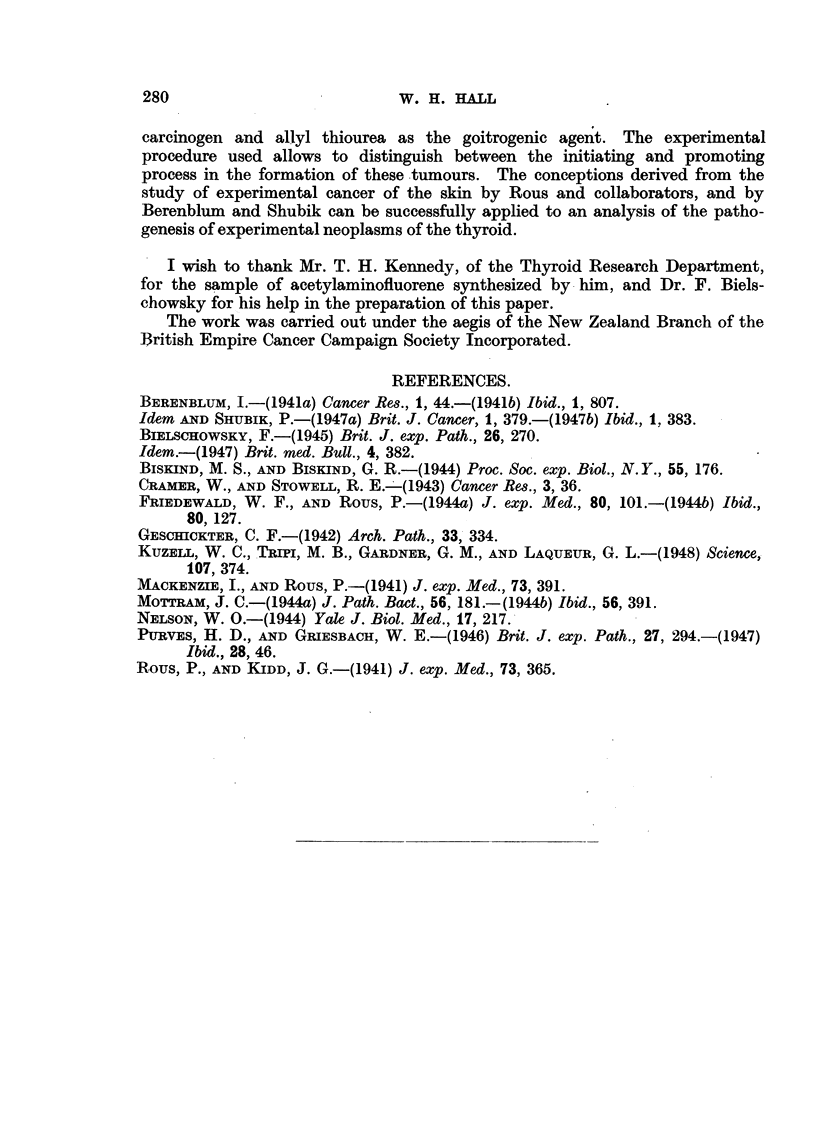# The Role of Initiating and Promoting Factors in the Pathogenesis of Tumours of the Thyroid

**DOI:** 10.1038/bjc.1948.32

**Published:** 1948-09

**Authors:** W. H. Hall

## Abstract

**Images:**


### THE ROLE OF INITIATING AND PROMOTING FACTORS IN THE PATHOGENESIS OF TUMOURS OF THE THYROID.

W. H. HALL.

*From the Cancer Research Department, University of Otago, Dunedin, N.Z.*

Received for publication July 6, 1948.

It has been realized for a long time that two processes might be involved in the pathogenesis of neoplasms, one which is concerned with the transformation of normal into neoplastic cells, and the other which promotes the growth of neoplastic cells into a visible tumour. Friedewald and Rous (1944*a*, 1944*b*) used the term "initiating" for the former, and "promoting" for the latter process. Rous and Kidd (1941), and MacKenzie and Rous (1941) analyzing the effects of tar and carcinogenic hydrocarbons on the skin of rabbits, found that these agents "cause many more cells to become tumour cells than give rise to visible growths." Berenblum (1941*a*) discovered that the yield of tumours of the skin induced in mice by sub-optimal doses of carcinogenic hydrocarbons could be increased by subsequent application of croton oil, and used the term "epi-carcinogenic action" to describe this effect. Mottram (1944*a*) refined Berenblum's technique; he found that tumours could be obtained by a single application of benzpyrene if the painting with the carcinogen was followed by repeated application of croton oil.. This has been confirmed by Berenblum and Shubik (1947*a*, 1947*b*).

It seemed important to investigate whether initiating and promoting factors were instrumental in the formation of tumours other than the skin. The thyroid was chosen as target organ and 2-acetylaminofluorene (A.A.F.) as the carcinogen. Bielschowsky (1945) obtained multiple adenomata of the thyroid by feeding A.A.F. to rats for 15 or 20 weeks, which was followed by administration of allyl-thiourea for up to 18 weeks. This experimental procedure did not allow a quantitative analysis of the factors involved in the pathogenesis of these thyroid tumours. Only by using small doses of A.A.F., which by itself are insufficient to induce neoplastic growth, could the role of initiating and promoting factors in the formation of multiple adenomata of the thyroid be ascertained.

### METHODS.

The rats used were females, aged 6 weeks, belonging to a strain of Wistar rats which had been imported into New Zealand 18 years ago. No foreign stock has been added to the colony during this period. The A.A.F. was given by stomach tube in a watery suspension. Each rat received four or six doses of 2·5 mg. of the carcinogen during the week. 4-methyl 2-thiouracil was administered as a 0·01 per cent solution in the drinking water. The daily average consumption was 8 c.c. of this solution. The rats were fed a dry diet consisting of kibbled maize and wheat supplemented by a mixture of bran 30 per cent, pollard 25 per cent, bone meal 15 per cent, pea meal 15 per cent, and maize meal 15 per cent. Thyroxine was given in the form of thyroid siccum (*B.P.*, 1932). Three

1 grain tablets of this product were ground and mixed with 1 kg. of the dry pulverized diet.

The thyroids were dissected out at autopsy and fixed in formol-saline. Serial sections were cut at 7 $\mu$ and stained with Harris's haematoxylin and eosin.

### EXPERIMENTAL.

Macroscopically the thyroids of the rats treated with A.A.F., and the glands of the controls receiving methyl thiouracil only, looked alike. In both groups the thyroid showed the typical picture of a hyperaemic goitre, the size of which increased progressively during the course of the experiment. However, the histological investigation of these glands revealed striking differences between the two groups (Fig. 1 and 2). As Table I shows, the rats in Group I*a*, which received 15 mg. of A.A.F. during the first week of the experiment, developed multiple adenomata. They were seen first in a rat killed 10 weeks after the withdrawal of the carcinogen, whereas an animal of the same group sacrificed 3 weeks earlier, showed only the well-known picture of hyperplasia and loss of colloid. The number and size of the adenomata increased during the course of the experiment, but their histological pattern remained essentially unaltered up to the twenty-first week, when the experiment was terminated. There was no indication of beginning malignancy, the nodules remaining sharply defined and showing orderly growth. Apart from the multiple adenomata of the thyroid, no other lesions were found which could be attributed to the action of A.A.F. Even when the duration of the experiment is prolonged for 18 months, the incidence of tumours induced by 10–15 mg. of A.A.F. in organs other than the stimulated thyroid is practically nil (to be published). The parathyroid glands were found to be rather large in all our rats receiving methyl thiouracil for prolonged periods. (This reaction of the parathyroid is under active investigation by Drs. W. E. Griesbach and J. Malcolm of the Medical School of Otago.)

Multiple adenomata were absent in the thyroids of all the controls (Table II), which were observed for 42 weeks. The only neoplastic changes seen in this group were single adenomata, the first of which was found in a rat which had received methyl thiouracil for 21 weeks. This was of minute size involving only a few follicles. In three of the four animals killed after 42 weeks of treatment with methyl thiouracil, single adenomata were also found. One of these was clearly recognizable at the post-mortem. Histologically it was a large cyst filled partly with blood, and partly with colloid ; in the periphery of the cyst adenoma there was a zone of smaller follicles which were distinct from the follicles of the rest of the gland (Fig. 3). Here the epithelium had nuclei which were richer in chromatin, giving this area a bluish appearance in the stained section.

---

### EXPLANATION OF PLATE.

FIG. 1.—Multiple adenomata (Rat 7, Group I*a*). × 11.

FIG. 2.—Simple hyperplasia (Rat 5, Group II). × 11.

FIG. 3.—Single adenoma (Rat 16, Group II). × 11.

FIG. 4.—Multiple cystic adenomata (Rat 10, Group I*b*). × 11.

FIG. 5.—Detail from Fig. 1. × 95.

FIG. 6.—Detail from Fig. 4. × 95.

Hall.

TABLE I.—*Occurrence of Thyroid Adenomata in Rats Treated First with A.A.F. and Subsequently with Methyl Thiouracil.*

| Group. | | Rat No. | Weeks of stimulation (methyl thiouracil). | Thyroid. |
|---|---|---|---|---|
| Ia | | 1 | 8 | Hyperplasia only. |
| | | 2 | 11 | Multiple adenomata. |
| | | 3 | 15 | ,, ,, |
| | | 4 | 19 | ,, ,, |
| | | 5 | 21 | ,, ,, |
| | | 6 | 21 | ,, ,, |
| | | 7 | 21 | ,, ,, |
| Ib | 4 weeks | 8 | 25 | ,, ,, |
| | ,, | 9 | 25 | ,, ,, |
| Thyroxine treated ; | 5 weeks | 10 | 26 | ,, ,, |
| | ,, | 11 | 26 | ,, ,, |

TABLE II.—*Occurrence of Thyroid Adenomata in Rats Treated only with Methyl Thiouracil.*

| Group. | Rat No. | Weeks of stimulation (methyl thiouracil). | Thyroid. |
|---|---|---|---|
| II (Controls) | 1 | 8 | Hyperplasia only. |
| | 2 | 11 | ,, ,, |
| | 3 | 15 | ,, ,, |
| | 4 | 19 | ,, ,, |
| | 5 | 21 | ,, ,, |
| | 6 | 21 | ,, ,, |
| | 7 | 21 | Minute single adenoma. |
| | 8 | 25 | Hyperplasia only. |
| | 9 | 25 | ,, ,, |
| | 10 | 26 | ,, ,, |
| | 11 | 26 | ,, ,, |
| | 12 | 26 | ,, ,, |
| | 13 | 42 | ,, ,, |
| | 14 | 42 | Single adenoma. |
| | 15 | 42 | ,, ,, |
| | 16 | 42 | Large single cystic adenoma. |

An investigation of the response of these multiple adenomata to the action of thyroxine, was undertaken at the suggestion of Dr. H. D. Purves of the Thyroid Research Department. Desiccated thyroid gland was given to 4 rats during the last 4 to 5 weeks of the experiment (Table I, Group I*b*), the administration of the methyl thiouracil being continued. Fig. 4 shows the effect of the thyroid medication. The multiple adenomata persist, but show a pattern which differs considerably from the one seen in animals of Group I*a*. Whereas in the latter the epithelium of the hyperplastic tissue as well as of the adenomata is high and colloid cysts are only occasionally present (Fig. 5), the glands of the rats treated with desiccated thyroid show quite a different picture. Here the gland is full of colloid and the epithelium is low throughout; the adenomata frequently contain colloid cysts lined by low-cuboid or flat epithelium (Fig. 6). There is no indication that the administration of thyroxine in the dose employed had an adverse effect on the benign tumours of the thyroid. No signs of regression or necrosis were found.

Identical results were obtained in a second experiment. Eleven rats (Table III) received first methyl thiouracil for 2 weeks and subsequently the same treatment as the animals of Group I. Again desiccated thyroid was fed to rats No. 8–11. The first rat was killed in the eleventh week of the experiment, i.e. 8 weeks after the carcinogen was withdrawn. No adenomata were seen on histological examination. The other 10 rats, killed between the twelfth and twenty-sixth week, all had multiple adenomata. There was no difference in the structure of the thyroid glands between Groups I and III.

TABLE III.—*Occurrence of Thyroid Adenomata in Rats Treated First with Methyl Thiouracil for 2 Weeks, and Subsequently with A.A.F. and Methyl Thiouracil.*

| Group. | | Rat No. | Weeks of stimulation (methyl thiouracil). | Thyroid. |
|---|---|---|---|---|
| III*a* | | 1 | 2 + 8 | Hyperplasia only. |
| | | 2 | 2 + 11 | Multiple adenomata. |
| | | 3 | 2 + 15 | ,, ,, |
| | | 4 | 2 + 19 | ,, ,, |
| | | 5 | 2 + 21 | ,, ,, |
| | | 6 | 2 + 21 | ,, ,, |
| | | 7 | 2 + 21 | ,, ,, |
| III*b* | 4 weeks | 8 | 2 + 25 | ,, ,, |
| | ,, | 9 | 2 + 25 | ,, ,, |
| Thyroxine treated; | 5 weeks | 10 | 2 + 26 | ,, ,, |
| | ,, | 11 | 2 + 26 | ,, ,, |

It seemed important to establish whether the susceptibility of the thyroid gland to the action of A.A.F. could be increased by pre-treatment with a goitrogenic agent. Dr. F. Bielschowsky has allowed me to include in this paper an experiment performed by him in 1945 which he has already mentioned (Bielschowsky, 1947).

Twenty female rats received daily 6 mg. of allyl thiourea for 18 weeks, and subsequently a daily dose of 4 mg. of A.A.F. for 25 weeks. Rats No. 14 and 15 (Table IV) were killed a few days after the withdrawal of the carcinogen. The remainder of the animals were killed when the presence of a tumour was suspected or when the animal appeared seriously ill.

TABLE IV.—*Occurrence of Thyroid Adenomata in Rats Treated with Allyl-Thiourea and Subsequently with A.A.F.*

| Rat No. | Duration of experiment (days). | Thyroid. | Cancer in other organs. |
|---|---|---|---|
| 1 | 469 | Normal | None. |
| 2 | 442 | Large single adenoma | Small intestine. |
| 3 | 461 | Two adenomata | ,, ,, uterus. |
| 4 | 454 | Normal | Liver, duct. acust. ext. |
| 5 | 461 | Single cystic adenoma | ,, ,, ,, ,, |
| 6 | 377 | Normal | Liver. |
| 7 | 356 | Large single adenoma | Duct. acust. ext. |
| 8 | 433 | Normal | None. |
| 9 | 433 | ,, | Liver. |
| 10 | 426 | ,, | ,, |
| 11 | 377 | Single adenoma | ,, |
| 12 | 468 | Large single cystic adenoma | ,, |
| 13 | 400 | Normal | Liver, small intestine |
| 14 | 316 | Single adenoma | None. |
| 15 | 302 | Normal | ,, |
| 16 | 412 | ,, | Duct. acust. ext. |
| 17 | 384 | Single cystic adenoma | ,, ,, ,, |
| 18 | 405 | ,, ,, ,, | Liver, lung (adenoma). |
| 19 | 451 | ,, ,, ,, | Duct. acust. ext. |
| 20 | 463 | ,, ,, ,, | Lung (adenoma). |

The liver of all animals contained cystic cholangiomata.

Although the time of stimulation was long, and the carcinogen was fed for nearly half a year, there was not the slightest indication of an increased susceptibility of the thyroid to the action of A.A.F. (Table IV). The neoplastic changes found were single adenomata of varying size. These benign neoplasms were indistinguishable from the ones which appear after prolonged stimulation with goitrogenic agents, and which persist after their withdrawal. Only in one animal (rat 3) two minute nodules were present in the same lobe. It is of interest that A.A.F. did not transform these benign structures into cancers.

To test for the existence of latent neoplastic cells in the thyroid the following experiment was set up. Fourteen rats received first 4 doses of 2·5 mg. of A.A.F. given by stomach tube, and 4 to 18 weeks later treatment with methyl thiouracil was started. Table V shows the results. There was no essential difference whether or not the stimulation of the thyroid started immediately after pretreatment with the carcinogen or whether an interval elapsed before methyl thiouracil was given. All the animals which received methyl thiouracil for 13 or more weeks developed multiple adenomata, even when the interval was extended for 18 weeks.

TABLE V.—*Occurrence of Thyroid Adenomata in Rats which Received First* 10 *mg. of A.A.F. and Subsequently Methyl Thiouracil After an Interval of* 4–18 *Weeks.*

| Rat No. | Interval (weeks). | Weeks of stimulation (methyl thiouracil). | Thyroid. |
|---|---|---|---|
| 1 | 4 | 11 | Multiple adenomata. |
| 2 | 4 | 26 | ,, ,, |
| 3 | 4 | 26 | ,, ,, |
| 4 | 6 | 13 | ,, ,, |
| 5 | 6 | 26 | ,, ,, |
| 6 | 6 | 26 | ,, ,, |
| 7 | 8 | 11 | Hyperplasia only. |
| 8 | 8 | 14 | Multiple adenomata. |
| 9 | 8 | 26 | ,, ,, |
| 10 | 10 | 11 | Hyperplasia only. |
| 11 | 10 | 13 | Multiple adenomata. |
| 12 | 10 | 26 | ,, ,, |
| 13 | 14 | 24 | ,, ,, |
| 14 | 18 | 27 | ,, ,, |

### DISCUSSION.

The work of Rous and his collaborators, of Mottram, and of Berenblum, has firmly established the existence of latent neoplastic cells in the skin of animals treated with a dose of a carcinogen which in itself is ineffective to induce visible tumours. The existence of such latent cells in mice was demonstrated by applying an unspecific irritant to the skin which had received a single dose of a carcinogenic hydrocarbon (Mottram, 1944*a*; Berenblum and Shubik, 1947*a*). There is excellent agreement between the results of Mottram, and of Berenblum and Shubik except in one point. Mottram (1944*b*) believed that a hyperplastic epidermis is more susceptible to the action of the carcinogen than the normal one. Berenblum (1941*b*), and Berenblum and Shubik (1947*a*) could not find any evidence for a sensitizing action of croton oil applied previous to the carcinogen to the skin. The results obtained in this laboratory agree with those of Berenblum and Shubik. There was no evidence that pre-treatment of the thyroid with a goitrogenic agent sensitized the gland to the subsequent action of A.A.F.

It could be shown that a small dose of A.A.F., which alone is incapable of inducing visible tumours in any part of the body, must nevertheless transform many normal into neoplastic cells. No other explanation accounts for the formation of multiple adenomata of the thyroid which develop after 11 or more weeks of administration of methyl thiouracil. These neoplastic cells, from which the multiple adenomata of the thyroid originate, remain dormant unless an appropriate stimulus is applied to the tissue harbouring them.

The thyrotropic hormone of the pituitary is the active stimulus when methyl thiouracil, thiourea and other so-called goitrogenic agents are administered. In the experiments reported here, the thyrotropic hormone plays the role which croton oil plays in the experiments of Berenblum and Shubik (1947*a*, 1947*b*); A.A.F. in the doses used, acts on the thyroid in the same way as a single moderate does of a carcinogenic hydrocarbon. There exists, however, one difference between the action of croton oil and the effects obtained with prolonged hormonal stimulation. Croton oil, however long applied to the skin of mice, produces only diffuse hyperplasia, but several examples are known where long continued stimulation by hormones leads to formation of benign and malignant tumours. For instance, treatment with oestrogens leads to cancer of the breast in rats (Geschickter, 1942; Nelson, 1944), and administration of goitrogenic compounds induces cancer of the thyroid after 20 or more months (Purves and Griesbach, 1946), benign tumours appearing earlier (Purves and Griesbach, 1947). Similarly in spayed mice, tumours of the ovary can be obtained by transplantation of this organ into the spleen. Here the gonadotropic hormones are the stimulating agents (Biskind and Biskind, 1944). The tumours due to excessive hormonal stimulation develop rather slowly, and their pathogenesis must be different from that of the tumours described in this paper. The single adenomata which, as Bielschowsky (1945) has already shown, develop after stimulation with thyrotropic hormone in absence of a chemical carcinogen, very rarely appear as quickly and never with the regularity of the multiple tumours initiated by A.A.F. Even after 42 weeks some of the controls are free of neoplastic changes, whereas every single rat which had received previously A.A.F. developed multiple adenomata in a much shorter period. The multiple adenomata of the thyroid which we obtained, developed at a time when, as the controls show, hormonal stimulation alone produced mainly hyperplasia. They seem to appear nearly as quickly as the papillomata of the skin in the experiments of Mottram (1944*a*) and Berenblum and Shubik (1947*a*, 1947*b*). Unfortunately it is necessary to kill the animals in order to demonstrate the presence of the adenomata of the thyroid, so that it is difficult to discover the very early lesions.

It seems, therefore, justified to assume that, in the experiments reported in this paper, the thyrotropic hormone plays the same role in the pathogenesis of the multiple adenomata of the thyroid as the croton oil in the pathogenesis of the papillomata of the skin. The fact that only benign tumours are induced in both sets of experiments strengthen the analogy, and it seems legitimate to disregard the late effects of hormonal stimulation in the interpretation of the results. It is worth while mentioning that the choice of method is of utmost importance in experiments designed to demonstrate the role of initiating and promoting factors. For instance, when methylcholanthrene is used in high concentrations a single application is sufficient to induce benign or malignant tumours (Cramer and Stowell, 1943). Also large doses of goitrogenic agents, as used by Kuzell, Tripi, Gardner and Laqueur (1948), seem to hasten the appearance of multiple adenomata of the thyroid.

### SUMMARY.

The induction of multiple adenomata of the thyroid by 10–15 mg. of acetylaminofluorene followed by administration of methyl thiouracil is described, confirming the results of Bielschowsky (1945) who used much larger doses of the carcinogen and allyl thiourea as the goitrogenic agent. The experimental procedure used allows to distinguish between the initiating and promoting process in the formation of these tumours. The conceptions derived from the study of experimental cancer of the skin by Rous and collaborators, and by Berenblum and Shubik can be successfully applied to an analysis of the pathogenesis of experimental neoplasms of the thyroid.

I wish to thank Mr. T. H. Kennedy, of the Thyroid Research Department, for the sample of acetylaminofluorene synthesized by him, and Dr. F. Bielschowsky for his help in the preparation of this paper.

The work was carried out under the aegis of the New Zealand Branch of the British Empire Cancer Campaign Society Incorporated.

### REFERENCES.

BERENBLUM, I.—(1941*a*) *Cancer Res.*, **1**, 44.—(1941*b*) *Ibid.*, **1**, 807.

*Idem* AND SHUBIK, P.—(1947*a*) *Brit. J. Cancer*, **1**, 379.—(1947*b*) *Ibid.*, **1**, 383.

BIELSCHOWSKY, F.—(1945) *Brit. J. exp. Path.*, **26**, 270.

*Idem.*—(1947) *Brit. med. Bull.*, **4**, 382.

BISKIND, M. S., AND BISKIND, G. R.—(1944) *Proc. Soc. exp. Biol., N.Y.*, **55**, 176.

CRAMER, W., AND STOWELL, R. E.—(1943) *Cancer Res.*, **3**, 36.

FRIEDEWALD, W. F., AND ROUS, P.—(1944*a*) *J. exp. Med.*, **80**, 101.—(1944*b*) *Ibid.*, **80**, 127.

GESCHICKTER, C. F.—(1942) *Arch. Path.*, **33**, 334.

KUZELL, W. C., TRIPI, M. B., GARDNER, G. M., AND LAQUEUR, G. L.—(1948) *Science*, **107**, 374.

MACKENZIE, I., AND ROUS, P.—(1941) *J. exp. Med.*, **73**, 391.

MOTTRAM, J. C.—(1944*a*) *J. Path. Bact.*, **56**, 181.—(1944*b*) *Ibid.*, **56**, 391.

NELSON, W. O.—(1944) *Yale J. Biol. Med.*, **17**, 217.

PURVES, H. D., AND GRIESBACH, W. E.—(1946) *Brit. J. exp. Path.*, **27**, 294.—(1947) *Ibid.*, **28**, 46.

ROUS, P., AND KIDD, J. G.—(1941) *J. exp. Med.*, **73**, 365.